# Mechanism-based nonopioid analgesic targets

**DOI:** 10.1172/JCI191346

**Published:** 2025-06-02

**Authors:** Xiangsunze Zeng, Rasheen Powell, Clifford J. Woolf

**Affiliations:** F.M. Kirby Center, Boston Children’s Hospital and Department of Neurobiology, Harvard Medical School, Boston, Massachusetts, USA.

## Abstract

Acute pain management has historically been dominated by opioids, whose efficacy is overshadowed by the risks of addiction, tolerance, and dependence, culminating in the global opioid crisis. To transcend this issue, we must innovate beyond opioid-based μ receptor treatments, identifying nonopioid analgesics with high efficacy and minimal adverse effects. This Review navigates the multifaceted landscape of inflammatory, neuropathic, and nociplastic pain, emphasizing mechanism-based analgesic targets tailored to specific pain conditions. We delve into the challenges and breakthroughs in clinical trials targeting ion channels, GPCRs, and other molecular targets. We also highlight the intricate crosstalk between different physiological systems and the need for multimodal interventions with distinct pharmacodynamics to manage acute and chronic pain, respectively. Furthermore, we explore emerging strategies, including gene therapy, stem cell therapy, cell type–specific neuromodulation, and AI-driven techniques for objective, unbiased pain assessment and research. These innovative approaches are poised to revolutionize pain management, paving the way for the discovery of safer and more effective analgesics.

## Overview

**Figure 1:**
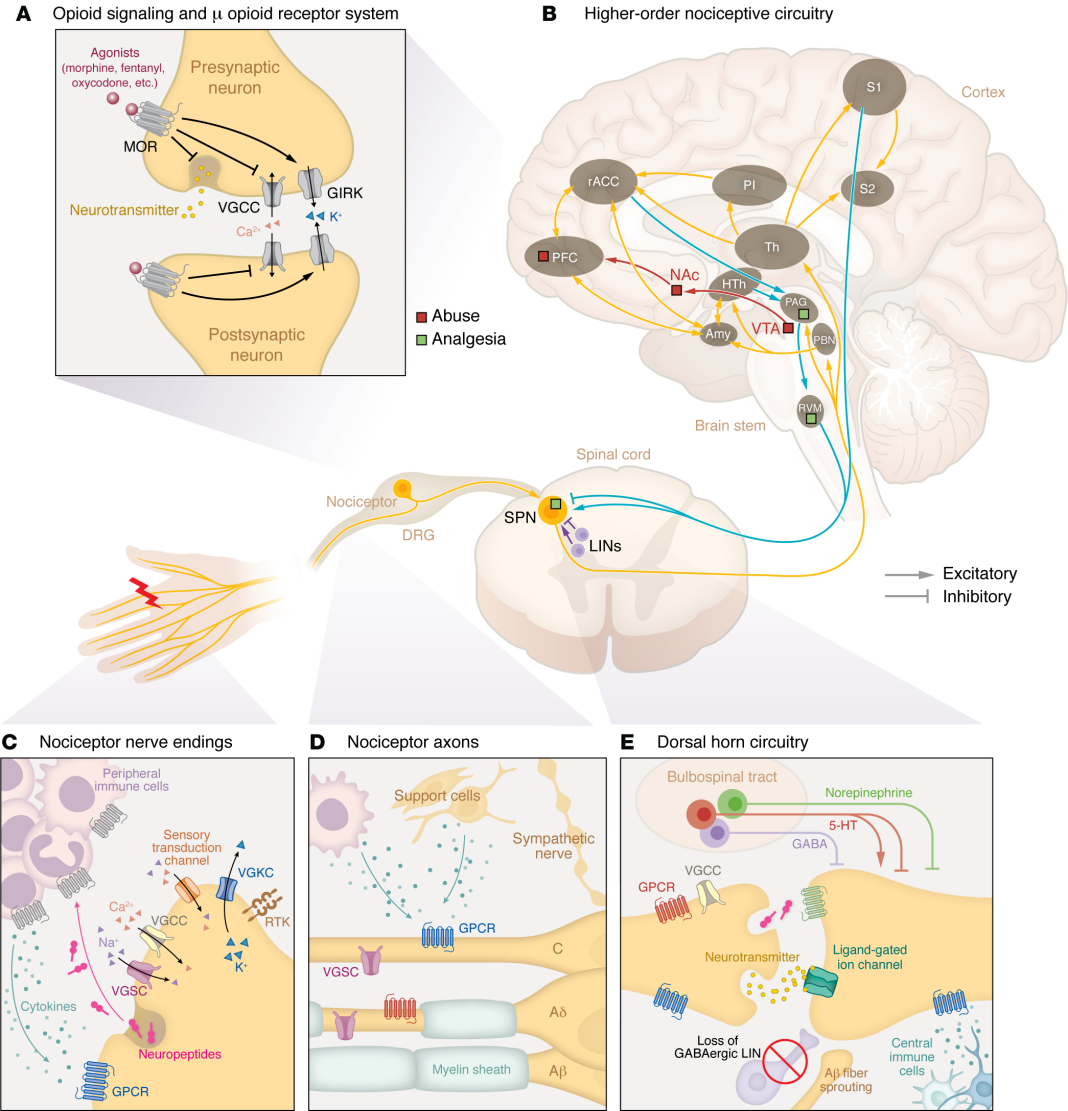
Molecular and circuit architecture of pain processing. (**A**) Activating the μ-opioid receptor (MOR) signaling system produces inhibitory effects on pain-initiating signal transmission but is also associated with adverse effects. See Table 1 for details. (**B**) Neural circuitry underlying nociceptive signal processing. Nociceptor afferents transmit signals from the periphery through the DRG to the spinal dorsal horn, where local interneurons (LINs) modulate the signals before relaying to higher-order brain structures via spinal projection neurons (SPNs). These structures include the parabrachial nucleus (PBN), periaqueductal gray (PAG), hypothalamus (HTh), thalamus (Th), prefrontal cortex (PFC), rostral anterior cingulate cortex (rACC), posterior insular cortex (PI), amygdala (Amy), and primary and secondary somatosensory cortices (S1, S2), constituting the ascending pathway (yellow). Descending pathways (blue) from the rACC, PAG, and rostral ventromedial medulla (RVM) modulate pain by inhibiting or facilitating spinal nociceptive transmission. The ventral tegmental area (VTA), nucleus accumbens (NAc), and PFC are implicated in the reward and abuse potential of opioids (red), whereas the PAG, RVM, and dorsal horn are primary sites for opioid-induced analgesia (green). (**C**) Peripheral tissue injury or pathogen invasion recruits immune cells that release proinflammatory cytokines, leading to heightened nociceptor excitability, which in turn drives neuropeptide release and amplifies inflammation. (**D**) Direct damage to nerves by injury or disease results in nociceptor hyperexcitability, demyelination, sympathetic nerve sprouting, and recruitment of peripheral immune cells to the site of injury that contribute to pain. Nonneuronal support cells secreting cytokines may exacerbate pain development. (**E**) Increases in ligand-gated ion channel activity, decreases in inhibitory GPCR signaling, loss of inhibitory LINs, and sprouting of nonnociceptive A fibers to the superficial dorsal horn can promote pain signaling. Recruitment of central immune cells (e.g., microglia and astrocytes) and a top-down regulation of serotonergic (5-HT), noradrenergic, and GABAergic projections via the bulbospinal tract also modulate CNS pain signals.

Effective pain management is crucial to improving the quality of life for people with acute and chronic pain. Traditional analgesics, like opioids, NSAIDs, serotonin-norepinephrine reuptake inhibitors (SNRIs), and gabapentinoids, are commonly used, but have profound limitations, including variable efficacy and adverse effects. Opioids that activate the μ receptors have long been the cornerstone in treating acute pain (Figure 1A). However, their use is hampered by high risk of abuse and dependency and both limited efficacy in managing chronic pain and serious adverse effects (1). The opioid crisis has had a devastating impact, with over 60 million people globally affected by opioid addiction and more than 100,000 annual opioid overdose deaths (2). These limitations underscore the urgent need for safer, more effective analgesics with minimal abuse potential.

## Addressing pain mechanisms before pain management

Acute nociceptive pain arises from the activation of nociceptors, which are high-threshold Aδ- and C-fiber sensory neurons innervating tissues to detect harmful/noxious stimuli. Nociceptors convert noxious stimuli into action potentials that propagate through the dorsal root ganglion (DRG) to the spinal dorsal horn, where the nociceptive input signal can be amplified or inhibited by local interneurons and descending regulation. The integrated signal is then relayed to the brain stem and higher-order brain regions, which process sensory-discriminative, affective, and cognitive dimensions of pain (Figure 1B) (3–6).

Under healthy conditions, pain is protective, serving as a vital warning system to detect danger and prevent injury from damaging stimuli, a process termed nociceptive pain. However, pain can transition into debilitating pathological forms due to inflammation, nerve damage, or dysregulated pain processing in the CNS. These conditions often present with hyperalgesia (heightened sensitivity to noxious stimuli), allodynia (pain in response to normally innocuous stimuli), and spontaneous pain. Effective management of pathological pain requires: avoiding reliance on opioid (specifically μ) receptor signaling (Figure 1, A and B); targeting specific regions and mechanisms (Table 1) underlying different pain conditions (Table 2); acknowledging the complex interactions among different biological systems; and preserving the fundamental protective elements of nociceptive function, especially in chronic pain conditions.

### Inflammatory pain.

Inflammatory pain arises from the immune system’s response to tissue injury, pathogen infections, or immune disorders (7). While inflammation was evolutionarily developed as a protective process — recruiting immune cells to eliminate pathogens, clear cellular debris, and promote wound healing — it can transition into persistent pain under chronic inflammatory conditions. Tissue damage activates the innate and adaptive immune systems, recruiting macrophages, mast cells, DCs, neutrophils, and T cells that release proinflammatory cytokines, chemokines, growth factors, and reactive oxygen species, many of which directly sensitize nociceptors by lowering their activation thresholds, causing hyperalgesia and allodynia (Figure 1C) (7). This peripheral sensitization, localized at the inflammation site, is a hallmark of inflammatory pain. Another important consideration is inflammatory priming, where an initial inflammatory insult “primes” nociceptors and immune cells, sensitizing them to subsequent stimuli (8). This priming may then lead to exaggerated painful responses to even mild inflammatory triggers (hyperalgesic priming), contributing to recurrent inflammatory pain (9).

Immune cells can exert both pronociceptive and antinociceptive effects. For example, macrophages activated by an acute injury or infection initially adopt proinflammatory phenotypes, releasing cytokines that sensitize nociceptors. Over time, they transition to antiinflammatory states, producing reparative mediators like IL-10 and thrombospondin-1, which counteract sensitization by modulating signaling pathways in peripheral terminals including PKA signaling (10). In a diet-induced diabetic-like neuropathy, macrophages recruited to the nerve help delay axonal degeneration (11). Managing inflammatory pain requires, therefore, understanding of the timing and context of the immune responses critical for determining the balance between pain sensitization and resolution. The goal is to selectively dampen pathological pain while preserving nociceptive protection and reparative immune functions.

### Neuropathic pain.

Neuropathic pain is caused by damage or disease affecting the somatosensory system. Unlike acute inflammatory pain, it is entirely pathological and typically persistent, lacks protective function, and is often accompanied by debilitating comorbidities like depression, anxiety, sleep disorders, and cognitive impairments (12–14). Spontaneous pain, a hallmark of neuropathic conditions, is more prevalent than stimulus-evoked pain (15). Mechanistically, neuropathic pain is multifaceted. Peripheral hyperexcitability due to aberrant ion channel expression, ectopic spontaneous discharges, and altered intracellular signaling pathways plays key roles (Figure 1D). Meanwhile, central sensitization enhances synaptic efficacy in the CNS through increased NMDA receptor activation and diminished GABAergic inhibition in the spinal dorsal horn (16–19). Structural plasticity, including synaptic reorganization, nonnociceptive Aβ fibers sprouting into central nociceptive pathways (20), activation of normally silent projection neurons (21), and expansion of peripheral nerve endings (22), extends hypersensitivity beyond the original injury site. Altered connectivity and plasticity in higher-order circuitry and bulbospinal descending control further exacerbate pain perception, reinforcing its chronic nature (Figure 1E).

“Neuropathic pain” encompasses a wide range of conditions with diverse etiologies, symptoms, and mechanisms (Table 2). Peripheral nerve injury–induced neuropathic pain involves aberrant axonal regeneration and spontaneous ectopic activity in injured axons (18, 19). By contrast, metabolic neuropathies, like diabetic peripheral neuropathy, arise from hyperglycemia-induced oxidative stress, resulting in nerve damage and pain (23). Similarly, neurotoxin-induced peripheral neuropathy, as seen in chemotherapy-induced peripheral neuropathy (CIPN), is associated with mitochondrial dysfunction and neurodegeneration (24). Conversely, central neuropathic pain, resulting from CNS damage, involves distinct mechanisms like loss of inhibitory control and cortical circuit reorganization (25). Neuropathic pain may cause paresthesia, dysesthesia, and itch, or negative symptoms like numbness and loss of touch or temperature sensations, reflecting its complex nature. The diverse etiologies highlight the need to contextualize neuropathic pain subtypes and identify specific pathophysiology to develop targeted therapies.

### Nociplastic pain.

“Nociplastic pain,” previously termed dysfunctional pain, describes conditions like fibromyalgia and complex regional pain syndrome type I (CRPS-I), where chronic pain occurs without apparent tissue damage or disease and is often accompanied by fatigue, sleep disturbances, cognitive impairments, and mood disorders (26). Patients often exhibit resistance to traditional analgesics like NSAIDs and opioids, while drugs targeting neuropathic pain, including gabapentinoids and SNRIs, offer only limited efficacy (27, 28). Consequently, first-line treatments prioritize nonpharmacological approaches like cognitive-behavioral therapy, given the strong psychological and emotional factors (29, 30). Nociplastic pain mechanisms remain the least understood and appear highly heterogeneous, primarily involving central sensitization (28), though peripheral alterations have also been suggested (31, 32), with genetic predisposition and environmental factors potentially also contributing to the development of nociplastic pain (33). Moreover, increased levels of proinflammatory cytokines (e.g., IL-6, IL-8, and TNF-α) are observed in both fibromyalgia and CRPS-I (34, 35), suggesting a potential immune contribution to the pathogenesis. These complexities, along with the lack of a well-defined etiology, make preclinical modeling challenging. Commonly used animal models (36, 37), like intramuscular acid injection, replicate only certain aspects of the clinical symptoms of fibromyalgia, leaving uncertainty whether they faithfully reproduce the pathophysiology.

The absence of reliable biomarkers further complicates diagnosis, and targeted therapies remain elusive. Current clinical studies largely rely on hemodynamic imaging (for example, functional MRI [fMRI]), leaving neural dynamics underexplored. Integrating complementary neuroimaging modalities to enhance spatiotemporal resolution, like fMRI-electroencephalography or -polysomnography fusion, alongside epigenetic and serological features (28, 33), could facilitate identification of bona fide biomarkers. Given the high prevalence of sleep disturbances in patients, potential biomarkers may also emerge during sleep (38). Furthermore, connectomics-based brain mapping (39) could refine pain classification for more precise diagnosis. These insights could inform the development of more physiologically relevant preclinical models to understand mechanisms, which are necessary to develop target-based therapies. Notably, a recent study showed that IgG transfer from fibromyalgia patients induces mechanical and thermal pain in mice, likely through binding to satellite glia and sensory neurons in the DRG, leading to nociceptor sensitization (40). This suggests immune components as viable therapeutic targets, and offers a more translational approach for modeling nociplastic pain.

### Multisystem involvement and multimodal intervention.

Pain is a complex sensory experience involving multiple systems. For instance, peripheral nerve injury from trauma triggers immune activation in several distinct ways. Initially, damage to nonneuronal tissues like skin recruits immune cells releasing proinflammatory mediators that sensitize nociceptors, causing an acute inflammatory component of the nerve injury pain (Figure 1, C and D). Persistent inflammation, however, may sustain pain, as seen in patients with chronic pain, in whom mast cell infiltration correlates with pain severity (41). Mechanistically, this may stem from inadequate production of antiinflammatory mediators like IL-4 and IL-10 (42), and/or prolonged T cell activation that amplifies pain signals (43). Conversely, the somatosensory system modulates immune function especially in chronic inflammation conditions like arthritis and colitis (44, 45). Neurons can interact directly with immune cells, as seen in a CIPN model where TLR on nociceptors activates immunity through MyD88 signaling (46). Additionally, sensory neurons release neuropeptides from their peripheral terminals, which act upon cognate receptors on immune cells (Figure 1C), leading to neurogenic inflammation (47). Calcitonin gene–related peptide (CGRP) released by nociceptors acts on bacteria-infected tissues to inhibit neutrophil recruitment (48), while the neurokinin-1 (NK1) receptor, activated by nociceptor-derived substance P, contributes to cutaneous inflammation (49). Similar phenomena are implicated in fibromyalgia (50). Such bidirectional neuron-immune communications can pathologically stabilize inflammatory and sensitized states, resulting in chronic pain. Beyond the periphery, injury or disease can drive CNS immune responses through microglia and astrocytes, further exacerbating central pain signaling (Figure 1E). However, the contribution of spinal microglial activation to neuropathic pain appears to be sexually dimorphic (51–53). Similarly, a recent study showed that meningeal regulatory T cells mediate a female-specific antinociceptive effect via δ-opioid receptors, a mechanism dependent on sex hormones rather than their canonical immune-regulatory role (54).

The autonomic system, particularly sympathetic nerves, contributes to neuropathic pain through signaling molecules like norepinephrine or by aberrant sprouting into the somatosensory system (55). In the same system, vascular movements have also been implicated in driving ectopic activity in the DRG via Piezo2 channels (56). Nonneuronal support cells including Schwann cells, satellite glia, and fibroblasts can promote pain development by secreting proinflammatory mediators and growth factors (Figure 1D) (57–60). Chronic pain may also affect the endocrine system, with hypothalamic-pituitary-adrenal axis dysregulation altering stress hormones like cortisol, affecting pain perception and inflammation as seen in fibromyalgia (61, 62). Similarly, in diabetes, hyperglycemia induces oxidative stress through the formation of glycation end products, which interact with neuronal and endothelial receptors, promoting nerve damage and neuropathic pain (63). Psychosocial factors also participate in shaping pain expression (64), with preclinical studies showing that pain-related behaviors can be socially transferred through higher-order cognitive circuitry, including the cingulate cortex (65).

The multisystem nature of pain underscores why therapies targeting only a single system often fail to fully alleviate the pain symptoms (66). This complexity emphasizes the need for multimodal approaches, either by combination of drugs that target a comprehensive spectrum of pathological changes across different physiological systems or by identification of a single compound that acts on multiple selected targets (67). While current clinical data suggest that such combinations often fail to produce synergistic effects (68), this could be due to the lack of mechanistic guidance on optimal drug pairings and the limitations of trial duration. By understanding the interplay among the engaged systems, future therapeutic strategies can better address the multifactorial nature of chronic pain, leading to more effective pain management.

### Considerations for treating acute and chronic pain.

Pain is a dynamic process that can transition from acute to chronic phases, each governed by distinct mechanisms. Acute pain typically results from the rapid peripheral sensitization of nociceptors due to inflammatory mediators released at the injury site. This sensitization enhances neuronal firing, leading to increased neurotransmitter release from the central terminals of nociceptors, which in turn amplifies pain signals to the brain. In contrast, chronic pain involves maladaptive plasticity within both the periphery and the CNS, often marked by irreversible physiological and anatomical changes. Notably, not all patients develop chronic pain, even after similar initial insults (69). This suggests that genetic and epigenetic factors drive long-term changes in gene expression that contribute to pain chronicity in susceptible individuals (70, 71). Critically, even if the pain primarily originates from one system, it can recruit other systems over time, evolving into a “mixed” pain condition. Persistent inflammation has been observed in chronic neuropathic conditions long after the initial trigger-induced inflammation has resolved (72). Likewise, while rheumatoid arthritis has a clear inflammatory origin, pain may persist despite eventual diminished tissue inflammation (66), with recent evidence suggesting that synovial fibroblasts contribute to pain persistence through interactions with sensory neurons (60).

**Figure 2:**
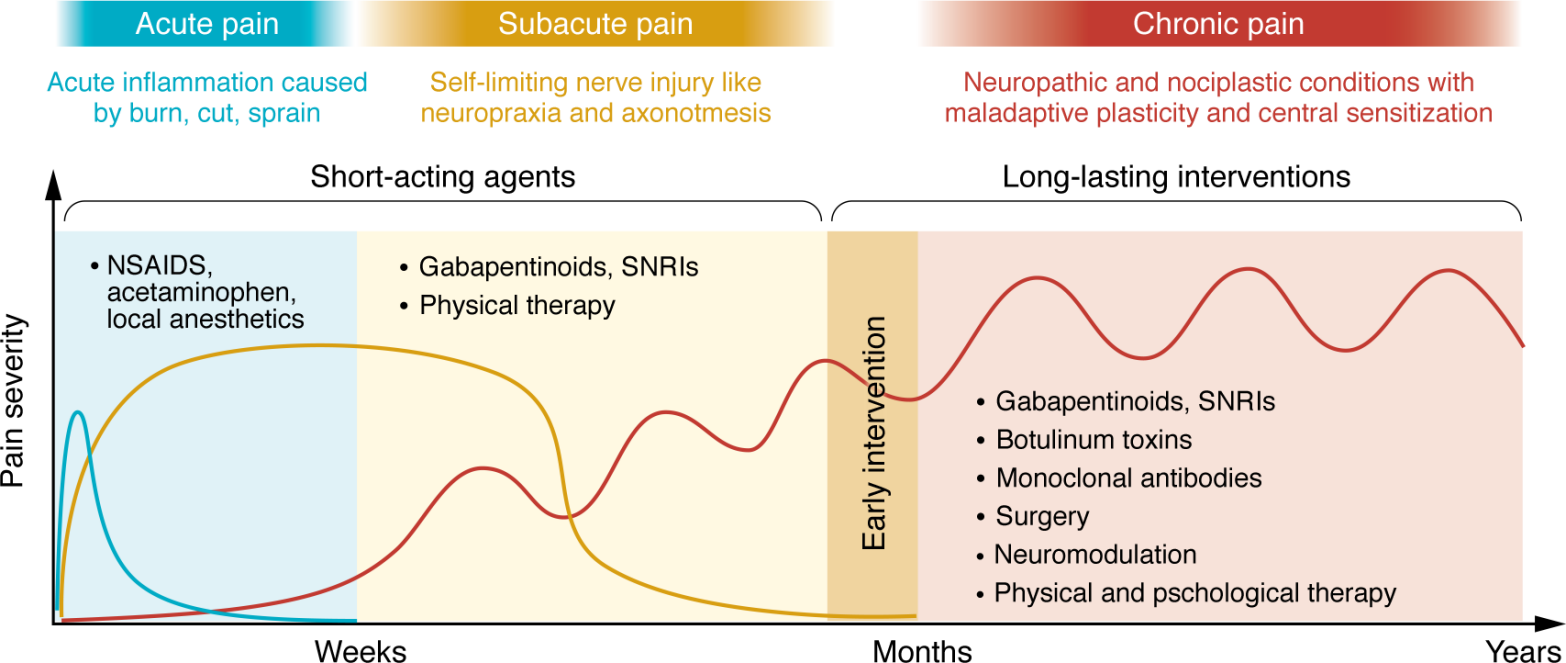
Acute and chronic pain demand distinct therapeutic strategies. Pain can be broadly categorized into acute, subacute, and chronic phases, each defined by distinct pathophysiological mechanisms and requiring tailored therapeutic strategies. This timeline illustrates the transition from short-acting symptomatic relief to more durable, disease-modifying interventions. Acute pain, typically mediated by nociceptor activation and inflammation, is commonly managed with short-acting agents such as NSAIDs, acetaminophen, and local anesthetics. Subacute pain — often resulting from injuries with regenerative potential, such as nerve compression (neuropraxia) or crush injuries (axonotmesis) — may resolve spontaneously and can be managed with gabapentinoids, SNRIs, and physical therapy. Chronic pain, particularly under neuropathic or nociplastic conditions (see Table 2), is frequently paroxysmal and recurrent, necessitating long-lasting interventions. Current options include pharmacotherapies, surgical interventions, neuromodulation, and multimodal physical and psychological therapies, though their efficacy remains limited and variable. Emerging approaches (see Figure 4B) may offer more targeted and sustained relief. Importantly, early interventions during the acute/subacute phase may help prevent the development of maladaptive plasticity, offering a neuroprotective strategy rather than merely suppressing symptoms once chronic pain is established.

The transition from acute to chronic pain also involves spatial shifts in pathophysiology. Following peripheral injury, central sensitization develops, while peripheral immune cells and proinflammatory cytokines may also infiltrate the CNS through a compromised blood-brain barrier (BBB), further amplifying pain signaling (43, 73). Understanding the spatiotemporal dynamics and mechanistic nature of pain progression is therefore crucial for effective intervention. We stress the need for longitudinal studies rather than heterogeneous patient cohorts to better capture pathological progression in individual patients. Furthermore, we must explore targeted therapies during the acute phase to prevent maladaptive changes from becoming entrenched, offering a neuroprotective strategy rather than merely suppressing established symptoms (Figure 2).

**Figure 3:**
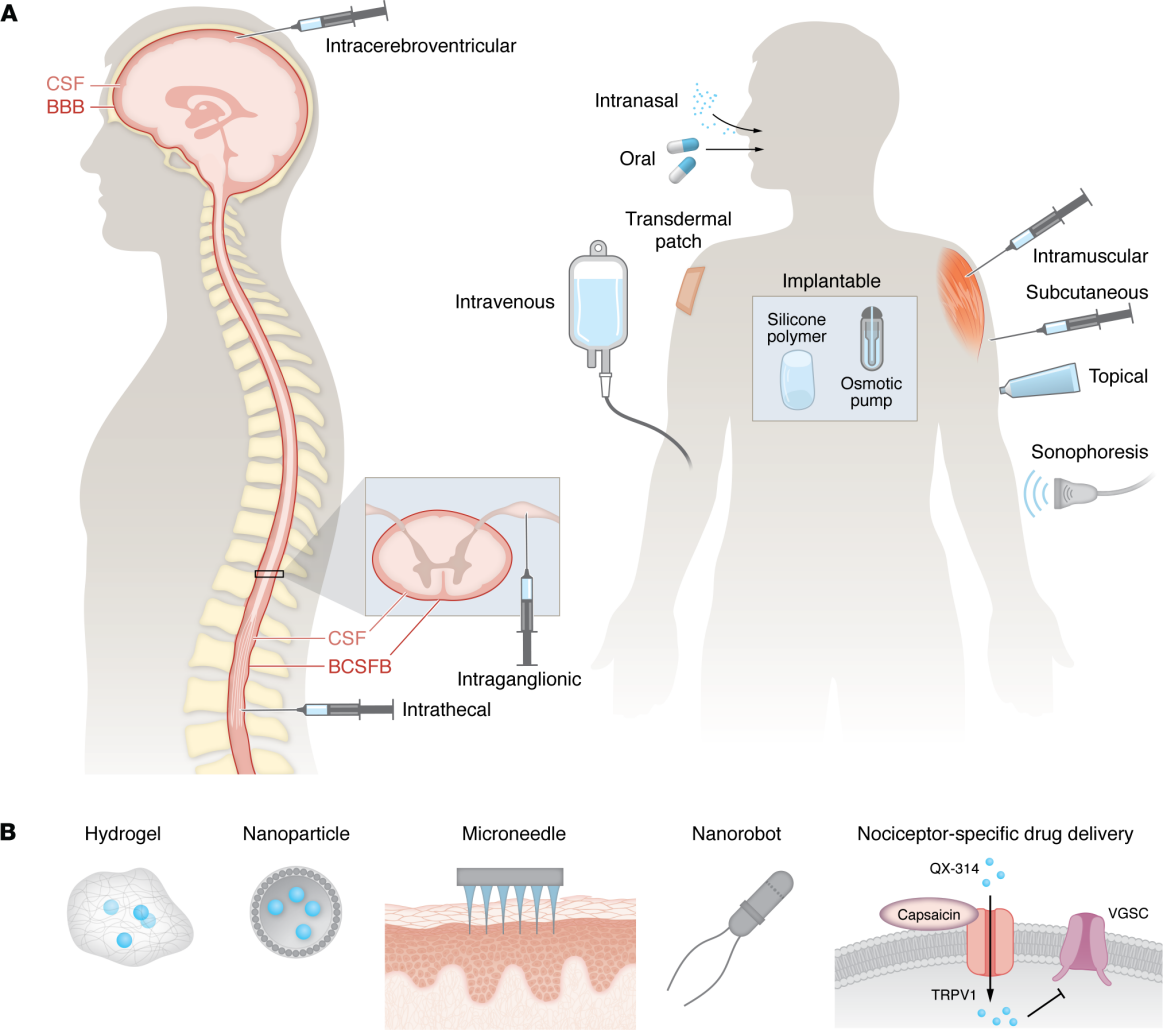
Drug delivery routes and techniques that enhance delivery efficacy and pharmacokinetics. (**A**) Oral administration is most common but suffers from first-pass metabolism and limited CNS penetration. Intranasal delivery offers rapid absorption via the olfactory and trigeminal pathways, enhancing brain access. Parenteral routes, including subcutaneous, intramuscular, and intravenous injections, provide faster onset but with systemic exposure and potential side effects. For localized pain control, topical and transdermal patch formulations minimize systemic effects while allowing sustained drug release. Sonophoresis enhances transdermal penetration using ultrasound waves (187). Intrathecal and intracerebroventricular delivery bypass the blood–cerebrospinal fluid barrier (BCSFB) and blood-brain barrier (BBB), allowing direct access to the cerebrospinal fluid (CSF). Intraganglionic administration is an effective route as the DRG is a primary site for the initiation of pain triggering signals and lies outside the BCSFB (188). Implantable systems, including silicone polymer–based depots and osmotic pumps, enable controlled, long-term drug release (189). (**B**) Biodegradable hydrogels offer sustained drug release, providing localized delivery with minimal systemic side effects (190). These hydrophilic networks respond to environmental triggers such as pH or temperature to control drug release (191). Nanoparticles, including lipid-based, polymeric, and inorganic variants, improve drug solubility, stability, and targeted tissue penetration while overcoming biological barriers (192). Engineered microneedles enable painless, transdermal drug delivery, bypassing the stratum corneum, improving bioavailability for molecules and biologics (193). Nanorobots, driven by magnetic, light, acoustic, or chemical propulsion, hold promise for precision-targeted drug delivery, actively navigating biological environments to reach specific tissues (194). There is also a targeted pain-specific local analgesia strategy involving coadministration of membrane-impermeant sodium channel blockers such as QX-314 or BW-031 with agonists that activate large-pore channels selectively expressed in nociceptors (e.g., capsaicin-TRPV1). This approach facilitates drug entry only into nociceptors, effectively blocking their activity while preserving motor and tactile function (195, 196).

Defining the underlying pathophysiology is essential for developing particular pharmacokinetic and pharmacodynamic strategies tailored to different pain states. For instance, postoperative pain or acute injury could be effectively treated with the oral or topical administration of short-acting antiinflammatory agents or ion channel blockers, while chronic pain with centralized components may require long-term targeted genetic modulation via neuraxial routes (Figure 2). Effective treatment depends not only on drug selection but also on delivery routes (Figure 3A), which influence drug bioavailability and target specificity. Despite efforts to develop more potent analgesics, their clinical efficacy remains limited if pharmacokinetic barriers exist, such as plasma protein binding and restricted BBB penetration. To overcome these challenges, a series of innovative technologies facilitating drug delivery have been developed (Figure 3B). For example, zinc and magnesium oxide nanoparticles exhibit intrinsic analgesic properties by depositing Zn^2+^ or Mg^2+^ ions into CNS synapses, thereby reducing ionotropic NMDA receptor activity (74, 75). Nanoparticle formulations of Zn^2+^ or Mg^2+^ also improve CNS penetration compared with ion administration alone. Additionally, nanoparticle-based carriers enhance the efficacy of local anesthetics, prolonging their duration and reducing systemic toxicity (76).

## Target-based pain management

In this section, we review molecular targets across anatomical sites for pain management (Table 1). The landscape of preclinical and clinical investigation is constantly advancing, and we have endeavored to highlight those select clinical trials that have resulted in promising clinical success or relative failure. We also discuss some promising targets being validated in preclinical research, as these may lay the groundwork for future clinical development. Clinical trial data were sourced from ClinicalTrials.gov and the International Clinical Trials Registry Platform, where we selected pain-related trials that targeted molecules including ion channels, GPCRs, enzymes, transporters, and others.

### Ion channels.

Voltage-gated sodium channels (VGSCs; referred to individually as Nav; Table 3) have been at the epicenter of drug design for the development of novel pain-killing drugs for several decades. Their role in membrane depolarization controls multiple aspects of neuronal excitability (i.e., action potential threshold, height, and width) (77). Furthermore, peripheral sensory neurons preferentially express a subset of VGSCs (Nav1.7, Nav1.8, and Nav1.9) that work in tandem to facilitate nociception (78). The importance of VGSCs in nociception is supported by the profound antinociception exerted by local anesthetics like lidocaine that nonselectively block VGSCs. However, their lack of subtype selectivity precipitates many undesired side effects, including loss of motor function and hypoesthesia. Therefore, considerable efforts have been expended to design potent analgesics with specificity for “nociceptive” VGSCs: Nav1.7, Nav1.8, and Nav1.9. So far, efforts have failed to develop a clinically viable molecule with subtype selectivity for VGSCs, with one notable exception: Vertex Pharmaceuticals was recently granted FDA approval for their selective Nav1.8 blocker suzetrigine (VX-548). The clinical success of suzetrigine provides evidence that Nav1.8 is a driver of pain and efficient blockade of Nav1.8 is sufficient in attenuating pain. However, the clinical trial data suggest that there is still room for improvement, as its greatest efficacy, which was limited, was observed in postsurgical pain (79) and diabetic neuropathy but not for sciatica, highlighting the importance of targeting the specific mechanisms underlying different pain conditions. On the other hand, TV-45070, a topical Nav1.7 blocker, has shown some promise, but failed to meet predetermined primary endpoints in postherpetic neuralgia patients (80, 81). Systemic administration of Nav1.7 blockers has failed to produce a clinical candidate either because of poor pharmacokinetics (82) or because they engaged Nav1.7 to produce analgesia but also precipitated effects on sympathetic neurons, leading to hypotension (83, 84). Curiously, a novel triple-acting (Nav1.7/1.8/1.9) molecule, ANP-230, has shown effectiveness in preclinical models in rodents (67, 85), which may be a useful strategy rather than targeting a single channel. Sodium channels are powerful determiners of nociceptor excitability; however, only relatively recently have we begun to tap into the clinical potential of selective VGSC blockade targeted at single or multiple specific subtypes. Ongoing efforts to explore other selective Nav inhibitors, some of which are actively recruiting participants for phase I/II clinical trials (86, 87), will hopefully result in an expansion of Nav channel inhibitors for treating pain. Selective Nav channel blockers may serve as key nonopioid pain relief options in the future.

The voltage-gated calcium channel (VGCC; referred to individually as Cav; Table 3) family represents a promising target for pain management, given its well-established role in regulating neuronal excitability and synaptic transmission (88). VGCCs are classified into 3 main types — Cav1 (L type), Cav2 (P/Q type, N type), and Cav3 (T type) — each playing distinct roles in neuronal function. Among these, the N-type calcium channel (Cav2.2) has garnered the most attention as a pain target due to its crucial role in mediating synaptic neurotransmitter release between primary sensory neurons and spinal dorsal horn neurons. A variety of ω-conotoxins have been developed to block Cav2.2 in preclinical pain models. Some, such as ziconotide, have reached the market. However, considerable drawbacks exist, particularly due to severe side effects like dizziness, nausea, and ataxia, as well as challenges in drug delivery methods, as intrathecal administration is required to bypass the BBB (89).

The calcium channel auxiliary protein α_2_δ subunit is also a major target for neuropathic pain. Trafficking of this auxiliary subunit is upregulated by axon damage, enhancing VGCC function, leading to increased neurotransmitter release and pain signaling (90, 91). Gabapentinoids (i.e., gabapentin and pregabalin) target the α_2_δ subunit and are widely used for neuropathic pain management with potential applicability for fibromyalgia (27, 91). However, responses vary substantially across individuals and pain conditions, and these medications are associated with cognitive and sedative adverse effects (92).

Potassium channels are responsible for potassium efflux resulting in membrane hyperpolarization, reducing neuronal excitability (93). Thus, enhancement of the efflux of potassium ions in nociceptors would be an attractive strategy for treating pain (94). There has been one notable success in this investigational landscape, flupirtine. Flupirtine is an aminopyridine that enhances currents through the voltage-gated potassium channels (VGKCs; referred to individually as Kv; Table 3) KCNQ (Kv7) and G protein–gated inwardly rectifying potassium (GIRK) channels and decreases currents through NMDA receptors (95, 96). The multimodal mechanism of action of flupirtine resulted in considerable analgesia in clinical trials across various pain modalities, contributing to its initial success (97, 98). However, severe hepatotoxicity led to its withdrawal from the market (99). Other potassium channel modulators being developed for epilepsy and ALS may have analgesic efficacy similar to that of flupirtine without its adverse effects (100).

Another straightforward strategy for alleviating pain triggered by external stimuli is to directly target molecular pain triggers, namely peripherally expressed sensory transduction channels (Table 3). Extensive preclinical research has focused on these channels, particularly the transient receptor potential (TRP) channel family (101) and mechanosensitive Piezo2 channels (102, 103). However, translating these findings into the clinic has proven challenging. Many of these channels are broadly expressed across various sensory cell types, meaning they are not exclusively linked to pain but are also essential for other physiological functions. Consequently, systemic inhibition can lead to side effects, and clinical success has been limited. One achievement is localized/topical modulation of TRPV1, which has shown efficacy in treating both inflammatory and neuropathic pain. Topical application mitigates the risk of off-target effects, preserving basal sensory functions while offering anatomically defined pain relief, an advantage over systemic administration, which can disrupt key protective functions of nociceptive pain and alter thermoregulation (101). It is important to recognize that distinct sensory cell types may contribute to different pain conditions, such that the precise targeting of specific cell populations (104) may be a useful strategy.

Postsynaptic ionotropic receptors (Table 3), NMDA, AMPA, and GABA type A (GABA_A_), are also options for pain management, as they play critical roles in processing and relaying pain-initiating signals from the spinal cord to higher-order circuits (5). Synaptic transmission involving these receptors produces both excitatory and inhibitory signals through glutamatergic and GABAergic synapses, respectively. Therefore, analgesia can be achieved through antagonizing excitatory (NMDA and AMPA) and agonizing inhibitory (GABA_A_) receptors, especially via the α_2_ subunit (105–107). These receptors are particularly pertinent in the context of inflammatory and neuropathic pain, where both sensitization and disinhibition in the CNS are key mechanistic features (16, 18, 19). However, owing to their broad involvement in general neurotransmission across the central and peripheral nervous systems, targeting these receptors for analgesia can lead to serious side effects, limiting their clinical applicability.

### GPCRs.

Among the myriad of receptors that populate nociceptor cell membranes, ion channels represent only a subset of pharmacologically exploitable molecules. Another major group are GPCRs, which transform extracellular stimuli into intracellular signaling cascades that modulate cellular responses depending on their associated G protein (Table 4). In neurons, strong changes in output, either excitatory or inhibitory, are typical following GPCR activation. GPCRs participate in nociceptive signal processing in varying capacities. From a preclinical perspective, there have been great advancements in understanding how they modulate pain signaling. For example, neuropeptide Y receptors 1 (NPY1R) and 2 (NPY2R) are Gi-coupled GPCRs that, in preclinical models, antagonize neuronal hyperexcitability in the spinal dorsal horn, reducing nociception (108, 109). However, clinical exploitation of these GPCRs has not been realized. This does not imply that NPYR1 and NPYR2 are not important for human nociception; rather, clinical data on these have yet to materialize. There have been some forays into GPCR modulation of pain in patients. Novartis Pharmaceuticals’ angiotensin II type 2 receptor antagonist EMA401 was developed to inhibit AGTR2, as AGTR2 was discovered to contribute to neuropathic pain (110). EMA401 was administered to participants with postherpetic neuralgia, who reported a decrease in their pain metrics; however, hepatotoxicity resulted in early termination of the trial (111). AstraZeneca developed a small-molecule negative allosteric modulator of the CCR2 receptor, AZD2423. Although it had high target engagement, there were no appreciable changes in reported pain scores in a double-blind placebo-controlled clinical trial (112). Pfizer attempted to leverage the modulation of nociceptive signaling with a cannabinoid receptor 2 (CB2) agonist, olorinab (ADP-371). CB2 agonism is a powerful modulator of nociceptive input into the spinal cord (113). In preclinical models of colitis, olorinab reversed abdominal hypersensitivity (114), which mirrored earlier reports of CB2-mediated analgesia in rodents. Unfortunately, olorinab did not reach the desired primary endpoint in a phase IIb clinical trial (115), although it did reduce average reported abdominal pain scores in patients with moderate to severe colitis-related pain.

Cannabis-based medicines, particularly cannabidiol (CBD) and tetrahydrocannabinol (THC), have been used for some neurological conditions (116) owing to their interactions with a wide variety of molecular targets. Preclinical studies indicate that THC exerts analgesia primarily through activation of the CB1 and CB2 GPCRs, while CBD modulates pain thorough a multitarget mechanism, including serotoninergic receptors (5-HT_1A_), TRP channels (117, 118), sodium channel blockade, and potassium channel activation (119, 120). Although there is some evidence for analgesic activity in chronic pain conditions, like fibromyalgia (121, 122), no cannabinoid-based therapies have been approved for pain management. Further studies with well-designed CBD/THC formulations or their analogs are needed to evaluate their therapeutic potential and long-term safety for pain management.

Serotonin and α_2_-adrenergic receptors are promising targets for pain modulation. 5-HT_1B/1D_ and 5-HT_1F_ receptor agonists are FDA-approved for acute migraine (123, 124), while a 5-HT_2_ receptor antagonist showed promise for fibromyalgia in a phase III trial (125). α_2_-Adrenergic agonists, including clonidine, dexmedetomidine, and tizanidine, offer strong analgesic/anesthetic effects, but clinical utility is limited by sedation and hypotension (126). Current efforts focus on subtype-selective adrenergic α_2_ agonists to minimize side effects. PS75, a functionally selective α_2A_ agonist, has analgesic efficacy with minimal sedation in animal models (127), suggesting potential for safe analgesic therapy.

### Enzymes, transporters, and others.

Enzymes and transporters have long been recognized for pain management (Table 5). NSAIDs like ibuprofen and aspirin are widely available over the counter because of their relatively safe profile at low doses. These drugs inhibit COX-2, an enzyme that produces prostaglandins, key mediators of inflammatory pain (128). Acetaminophen, a widely used, nonabusable painkiller, is a weak inhibitor of COX-2 in the CNS (129), but whether this is responsible for how it alleviates pain is uncertain. While COX-2 inhibitors are effective for many mild acute pain conditions, they exhibit limited efficacy in neuropathic and nociplastic pain, suggesting that these conditions involve prostaglandin E_2_–independent mechanisms. Antidepressants and SNRIs represent another traditional pain therapeutic that acts on a broad spectrum of molecules, modulating descending and ascending aminergic pain pathways. They show efficacy in some neuropathic and nociplastic conditions, especially those with strong centralized components (130). However, systemic delivery often causes off-target effects, and their efficacy varies across pain subtypes and individuals. Improving target specificity and minimizing adverse effects is critical to advancing pain management beyond these traditional pharmacotherapies.

Monoclonal antibody (mAb) therapies (Table 5) represent a potential transformative approach for pain management by targeting the immune-somatosensory interactions underpinning many pain conditions as discussed above. Unlike conventional drugs, mAbs offer unparalleled specificity and extended half-life, which minimizes off-target effects and enables sustained therapeutic effects with less frequent dosing (131). In preclinical models, anti-NGF antibodies reduce pain in cancer pain models in rodents (132). One anti-NGF antibody produced by Regeneron Pharmaceuticals, fasinumab, had clinical success in two separate trials in patients with low back pain and osteoarthritic knee pain (133, 134). However, treatment-associated adverse joint events were observed in participants with knee osteoarthritis (133). Another example is the CCL17 inhibitor GSK3858279 developed by GlaxoSmithKline. Instead of direct modulation of CCL17’s cognate receptor CCR4, GSK3858279 binds CCL17, preventing CCL4/CCL17-mediated immune cell activation, thus reducing pain (135, 136). In a phase I clinical trial for safety and efficacy in patients with knee osteoarthritis, weekly administration of GSK3858279 was well tolerated and significantly decreased pain scores (137), though in another trial in healthy participants, GSK3858279 did not reach desired primary endpoints (138), displaying that GSK3858279’s efficacy is dependent on the presence of a preexisting chronic inflammatory pain state. Additional efforts include AbbVie’s lutikizumab, an anti–IL-1α/β antibody. Like anti-NGF therapies, lutikizumab, a dual–variable domain immunoglobulin, was expected to bind and sequester IL-1α/β, reducing proinflammatory signaling and pain in inflammatory pain conditions (139, 140). In a phase II clinical trial in knee osteoarthritis, lutikizumab was, however, unable to significantly impact either the joint inflammation or its associated pain (141).

Although mAbs have shown promising clinical applications, particularly for anti-CGRP antibodies for treating migraine (142), challenges remain. Most mAbs are unable to cross the BBB, and their efficacy in treating neuropathic and nociplastic pain remains underexplored. Additionally, high production costs and the requirement for parenteral administration present logistical and economic barriers.

Another consideration for treating neuropathic pain is targeting not receptors but epigenetic factors that impact gene expression. Genetic reprogramming occurs in neurons along the “pain pathway” following a neuropathic insult (70, 71), and antagonizing this process may reduce pain. Inhibition of histone deacetylases (HDACs) prevented genetic repression in preclinical models of neuropathic pain and reduced pain hypersensitivity, suggesting that genetic expression changes may contribute to the initiation of neuropathic pain (143, 144). Regency Pharmaceuticals tested this concept using the HDAC6 inhibitor ricolinostat in patients with painful diabetic neuropathy (145). However, the HDAC6 inhibition did not significantly decrease patient pain scores after 12 weeks of treatment.

## Emerging novel technologies for pain management

**Figure 4:**
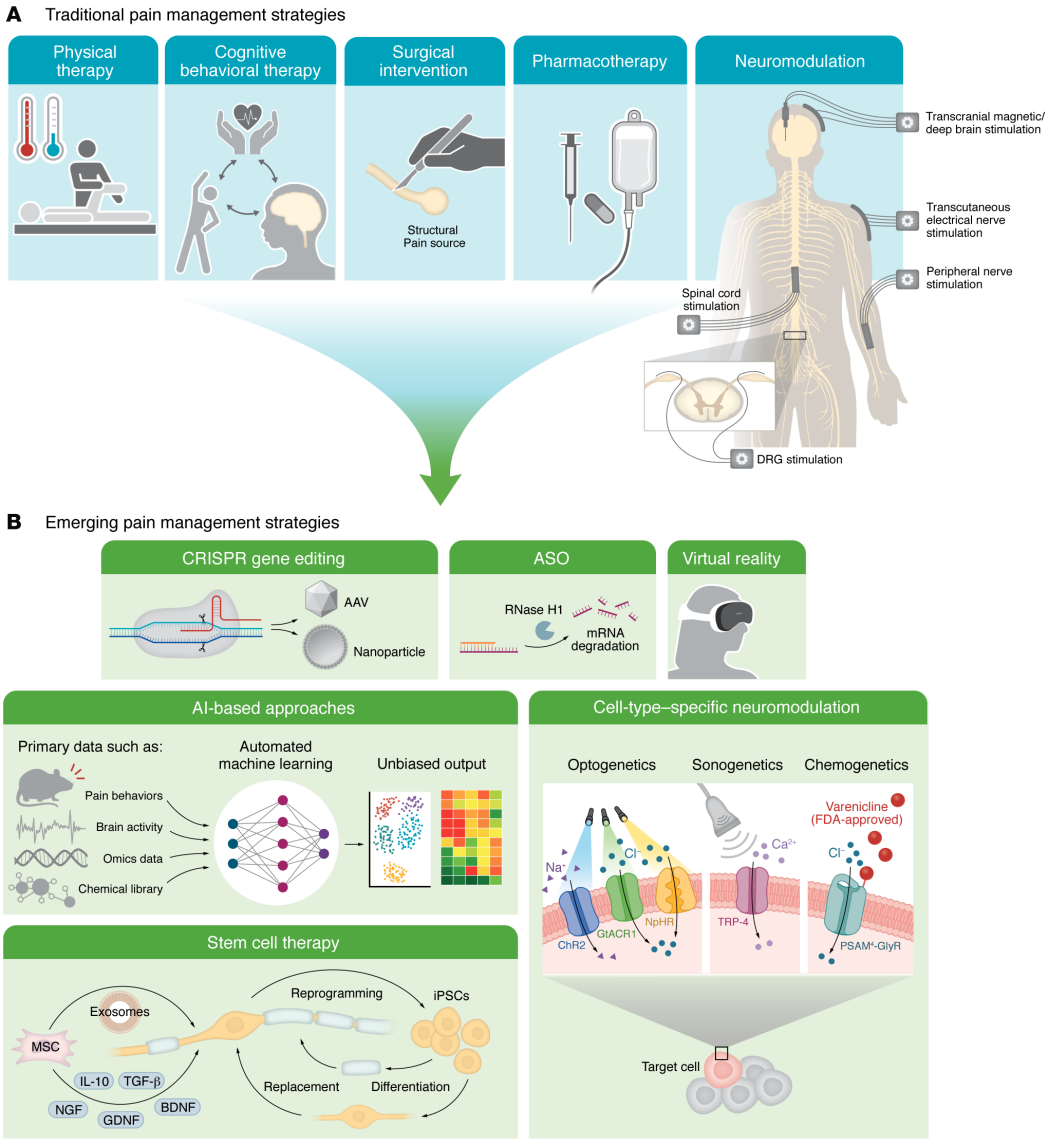
Current and emerging technologies for pain management. (**A**) Traditional approaches encompass pharmacotherapy, physical therapy (manual therapy, cryo-/thermotherapy), psychotherapy, surgery, and electrical neuromodulation, which are selected based on specific pain conditions. Physical and psychological (cognitive-behavioral therapy) therapies are often recommended for conditions resistant to conventional pharmacotherapy, like fibromyalgia (26). Surgical excision may address structural pain sources such as neuromas (197). Neuromodulation is typically reserved for refractory chronic pain unresponsive to standard treatments (198). In practice, multimodal approaches combining several strategies are common. (**B**) Emerging approaches aim to offer tailored, mechanism-based pain relief. CRISPR delivered through an adeno-associated virus (AAV) or nanoparticle allows precise editing of “pain genes” at the DNA level for permanent effects. Antisense oligonucleotides (ASOs) are short, single-stranded DNA or RNA strands that bind to specific mRNA transcripts, either degrading them via RNase H–mediated cleavage or blocking their translation, thereby transiently preventing production of pain-related proteins. Stem cell therapy using mesenchymal stem cells (MSCs) promotes tissue repair and reduces inflammation by secreting neurotrophic factors (NGF, GDNF, BDNF) and antiinflammatory cytokines (IL-10, TGF-β). MSC-derived exosomes may also serve as natural nanocarriers for delivering drugs or siRNAs (199). Patient-derived induced pluripotent stem cells (iPSCs) can be differentiated into DRG neurons and Schwann cells to repair or replace damaged tissues. Advanced neuromodulation leverages cell-specific genetic tools. Optogenetics can directly modulate neuronal activity with various opsins responsive to light of different wavelengths, inducing excitatory (ChR2) or inhibitory (GtACR1 or NpHR) effects. Sonogenetics couples ultrasound stimulation with mechanosensitive channels such as TRP-4, offering noninvasive deep tissue neuromodulation. Humanized PSAM^4^-GlyR chemogenetics using an FDA-approved agonist offers translational promise. AI/ML techniques not only enable automated unbiased analysis of pain behaviors, neuroimages, neural activity, and omics integration, but also advance drug discovery, and the modeling of cellular and circuit pain processes via AI-powered virtual cells (AIVCs). Finally, virtual reality that engages sensory and cognitive pathways can be an adjunctive therapy for certain chronic pain (200), like complex regional pain syndrome (CRPS) (ClinicalTrials.gov NCT04849897).

While various conventional approaches to pain management are available depending on specific pain contexts (Figure 4A), emerging technologies are revolutionizing the way we can approach mechanism-based pain treatment (Figure 4B).

Gene therapy using CRISPR-based techniques (146) or antisense oligonucleotides (ASOs) (147) is being explored to directly modulate pain-associated genes such as *SCN9A* (encoding Nav1.7) and *KCNA2* (Kv1.2) for targeted pain relief in preclinical models (148–150). CRISPR genomic editing can potentially provide a long-lasting effect after a one-time intervention, which may be particularly beneficial for refractory chronic pain conditions with strong genetic components. In contrast, ASOs act at the RNA level, allowing a reversible and tunable modulation for acute pain (Figure 2). While CNS delivery remains a major challenge, advancements in viral vectors and lipid nanoparticles (146) are bringing them closer to clinical pain management.

Stem cell therapy, unlike traditional strategies that mainly manage symptoms, offers an opportunity to address the root cause of pathological conditions like traumatic injuries by repairing damaged tissues. Mesenchymal stem cells have shown therapeutic benefits in conditions including spinal cord injury, chronic low back pain, and diabetic neuropathy, owing to their antiinflammatory and neurotrophic properties (151). Early proof-of-concept work is also exploring direct replacement of damaged sensory neurons using organoids derived from induced pluripotent stem cells (iPSCs) (152), often combined with bioengineered scaffolds like hydrogels to enhance integration and regeneration (153).

Advanced neuromodulation techniques, such as optogenetics and sonogenetics, are valuable for studying pain mechanisms and developing new treatments (154–157). Unlike conventional electrical neuromodulation, which lacks cell type specificity, they offer superior spatiotemporal precision by using light or ultrasound paired with targeted genetic tools. However, clinical translation faces major challenges, including efficient delivery of opsins to human neurons and the need for advanced optics to reach deep tissues. While sonogenetics allows deeper tissue penetration, its reliance on mechanosensitive proteins raises concerns about off-target effects from endogenous channel expression. Nevertheless, progress in viral vectors and gene-editing technologies is narrowing the gap to clinical applications. Notably, a humanized chemogenetic system, PSAM^4^-GlyR (158), has been recently characterized, which offers a greater translational potential over conventional Designer Receptors Exclusively Activated by Designer Drugs (DREADDs), with faster chloride channel conductance, all-human receptor components, and an FDA-approved agonist, varenicline.

The emergence of artificial intelligence–based (AI-based) methods is transforming pain research by enabling precise diagnosis, biomarker identification, and the discovery of novel therapies. In preclinical research, machine learning–based (ML-based) techniques such as DeepLabCut (159) have been applied to objectively phenotype pain behaviors (160, 161). Unsupervised algorithms like Motion Sequencing (MoSeq) are also emerging to uncover hidden behavioral patterns invisible to human observation (162, 163). In clinical settings, ML is increasingly used to identify potential biomarkers from neuroimaging data and brain activity recorded from patients with chronic pain (164, 165). ML further aids in analyzing transcriptomic (166–169), proteomic (170, 171), and interactomic (172–174) datasets and GWAS (175, 176) to identify context-specific pain-related genes and pathways. Moreover, AI supports drug development and identification of novel analgesic compounds through virtual screening and de novo molecular design (177, 178). Emerging tools such as AI-powered virtual cells (AIVCs) (179) simulate nociceptor function, circuit connections, and neuroimmune crosstalk, allowing prediction of analgesic efficacy in silico. These innovations accelerate pain research by bridging computational models and experimental studies to guide pain management strategies, though the need for high-quality, representative datasets, and the risk of overfitting or poor generalizability, remain issues that must be addressed.

## Charting the future: pathways forward

While many promising targets have been or are being identified for pain management, fewer than 10% of candidate drugs gain approval (180). This high failure rate is largely due to the limited efficacy in humans compared with animal models, severe adverse events, and poor pharmacokinetics (181). Addressing these challenges requires a comprehensive understanding of the molecular, cellular, and circuit mechanisms underlying each pain condition and differentiating pain modalities (e.g., thermal, mechanical, and spontaneous pain). A deeper understanding of species differences, including insights from studies using primary human DRGs (52, 168, 182), is crucial to improve the translational relevance and impact of preclinical studies. Additionally, sex differences in pain mechanisms, particularly in conditions with pronounced bias such as fibromyalgia, might necessitate sex-specific therapeutic strategies (51–54, 183). Developing preclinical models that closely replicate human pain conditions is essential, as many current models fail to capture the complexity of clinical pain — particularly its chronic nature. This includes utilizing in vivo longitudinal imaging approaches to investigate behavioral and neuronal changes (21, 22, 184), and in vitro modeling using patient-derived iPSCs to generate human sensory neurons and CNS organoids (185, 186), capturing key aspects of individual susceptibility to chronic pain development. For clinical research, it is vitally important to improve trial design, as limitations including underpowered studies, short trial durations that fail to capture the chronic features, and mismatches between preclinical and clinical conditions (e.g., testing of a drug validated in traumatic injury on diabetic neuropathy patients) can profoundly impact outcomes. It is also important to adopt advanced imaging techniques and ML-based phenotypic approaches to identify precise biomarkers for pain diagnosis and treatment rather than relying only on patient self-reporting. This is especially critical for nonverbal populations, including infants and individuals with cognitive impairments. By integrating cutting-edge technologies, refining preclinical models, and enhancing clinical methodologies, the field can begin to address and overcome key barriers more effectively to improve pain management, which could pave the way for safer, more effective, and personalized precision therapies, as well as lead to the elimination of prescription opioids.

## Figures and Tables

**Table 5:**
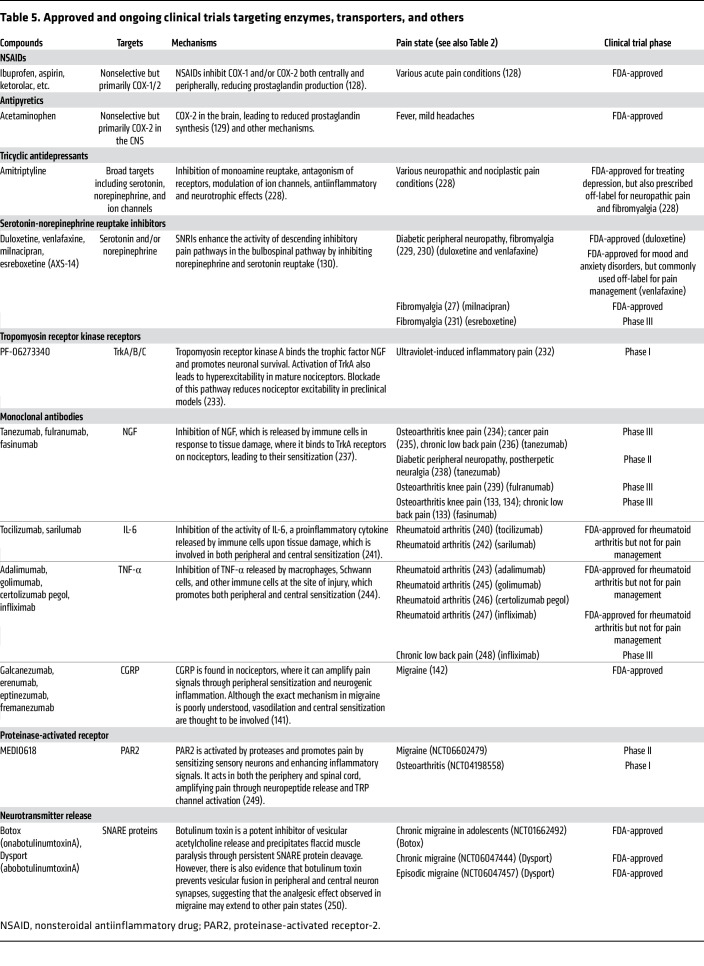
Approved and ongoing clinical trials targeting enzymes, transporters, and others

**Table 4:**
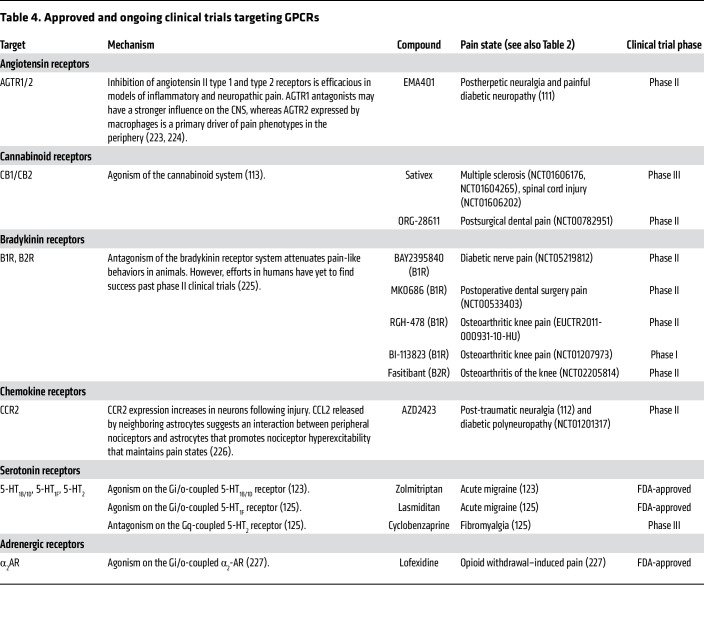
Approved and ongoing clinical trials targeting GPCRs

**Table 3:**
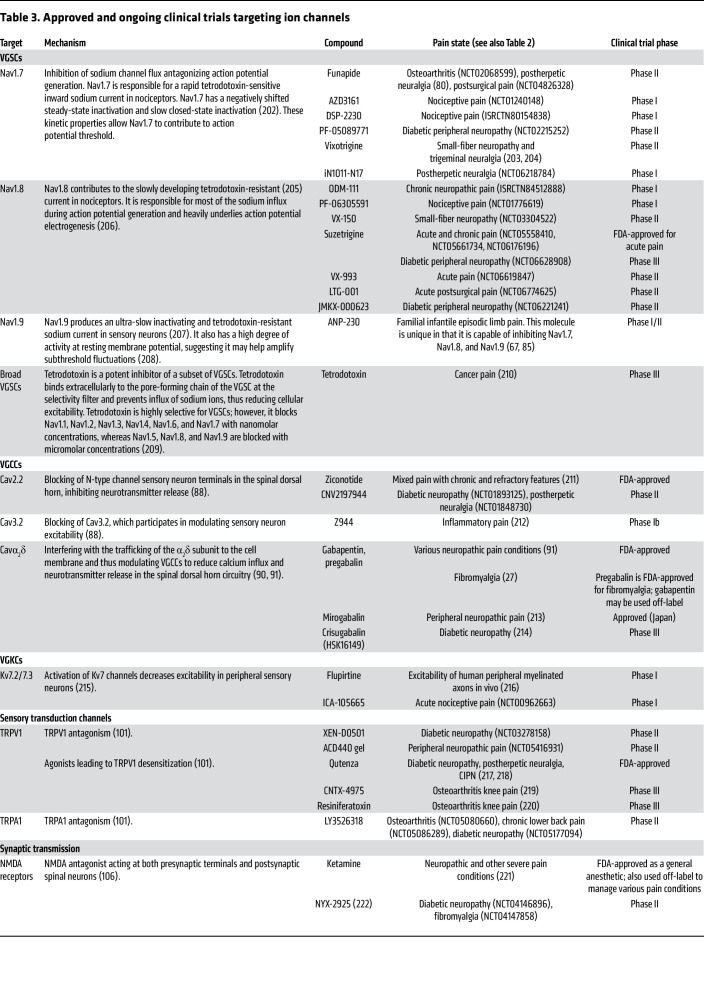
Approved and ongoing clinical trials targeting ion channels

**Table 2:**
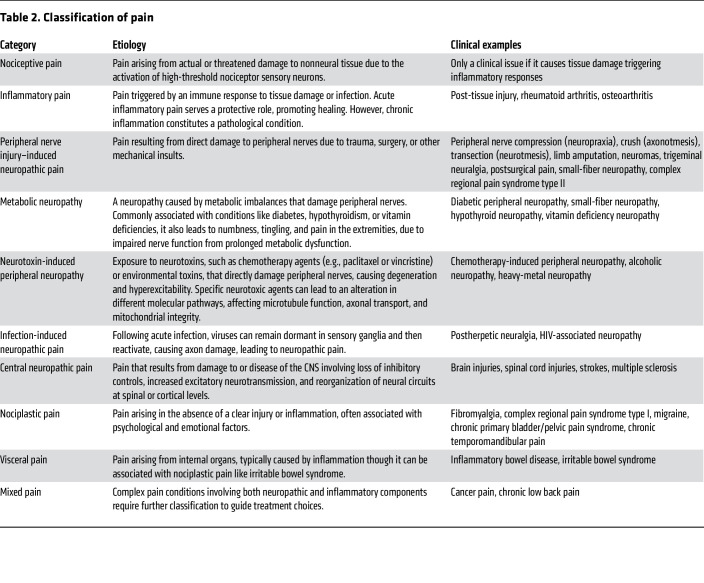
Classification of pain

**Table 1:**
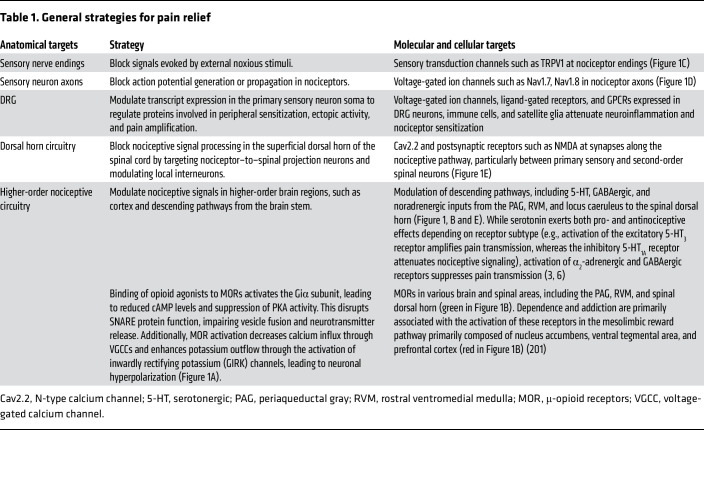
General strategies for pain relief

## Version 1 06/02/2025

Electronic publication

## References

[1] Benyamin R (2008). Opioid complications and side effects. Pain Physician.

[2] The Lancet Regional Health-Americas (2023). Opioid crisis: addiction, overprescription, and insufficient primary prevention. Lancet Reg Health Am.

[3] Kuner R, Kuner T (2021). Cellular circuits in the brain and their modulation in acute and chronic pain. Physiol Rev.

[4] Basbaum AI (2009). Cellular and molecular mechanisms of pain. Cell.

[5] Todd AJ (2010). Neuronal circuitry for pain processing in the dorsal horn. Nat Rev Neurosci.

[6] Ossipov MH (2010). Central modulation of pain. J Clin Invest.

[7] Talbot S (2016). Neuroimmunity: physiology and pathology. Annu Rev Immunol.

[8] Flayer CH (2024). A γδ T cell-IL-3 axis controls allergic responses through sensory neurons. Nature.

[9] Kandasamy R, Price TJ (2015). The pharmacology of nociceptor priming. Handb Exp Pharmacol.

[10] Hakim S (2024). Immune drivers of pain resolution and protection. Nat Immunol.

[11] Hakim S (2025). Macrophages protect against sensory axon loss in peripheral neuropathy. Nature.

[12] Nicholson B, Verma S (2004). Comorbidities in chronic neuropathic pain. Pain Med.

[13] Alexandre C (2024). Nociceptor spontaneous activity is responsible for fragmenting non-rapid eye movement sleep in mouse models of neuropathic pain. Sci Transl Med.

[14] Baron R (2010). Neuropathic pain: diagnosis, pathophysiological mechanisms, and treatment. Lancet Neurol.

[15] Bouhassira D (2004). Development and validation of the neuropathic pain symptom inventory. Pain.

[16] Woolf CJ (2011). Central sensitization: implications for the diagnosis and treatment of pain. Pain.

[17] Moore KA (2002). Partial peripheral nerve injury promotes a selective loss of GABAergic inhibition in the superficial dorsal horn of the spinal cord. J Neurosci.

[18] Costigan M (2009). Neuropathic pain: a maladaptive response of the nervous system to damage. Annu Rev Neurosci.

[19] Finnerup NB (2021). Neuropathic pain: from mechanisms to treatment. Physiol Rev.

[20] Woolf CJ (1992). Peripheral nerve injury triggers central sprouting of myelinated afferents. Nature.

[21] Yarmolinsky DA (2025). Differential modification of ascending spinal outputs in acute and chronic pain states. Neuron.

[22] Gangadharan V (2022). Neuropathic pain caused by miswiring and abnormal end organ targeting. Nature.

[23] Singh R (2014). Diabetic peripheral neuropathy: current perspective and future directions. Pharmacol Res.

[24] Zajaczkowska R (2019). Mechanisms of chemotherapy-induced peripheral neuropathy. Int J Mol Sci.

[25] Rosner J (2023). Central neuropathic pain. Nat Rev Dis Primers.

[26] Fitzcharles MA (2021). Nociplastic pain: towards an understanding of prevalent pain conditions. Lancet.

[27] Tzadok R, Ablin JN (2020). Current and emerging pharmacotherapy for fibromyalgia. Pain Res Manag.

[28] Siracusa R (2021). Fibromyalgia: pathogenesis, mechanisms, diagnosis and treatment options update. Int J Mol Sci.

[30] Harden RN (2022). Complex regional pain syndrome: practical diagnostic and treatment guidelines, 5th edition. Pain Med.

[31] Serra J (2014). Hyperexcitable C nociceptors in fibromyalgia. Ann Neurol.

[32] Uceyler N (2013). Small fibre pathology in patients with fibromyalgia syndrome. Brain.

[33] D’Agnelli S (2019). Fibromyalgia: genetics and epigenetics insights may provide the basis for the development of diagnostic biomarkers. Mol Pain.

[34] Mendieta D (2016). IL-8 and IL-6 primarily mediate the inflammatory response in fibromyalgia patients. J Neuroimmunol.

[35] Parkitny L (2013). Inflammation in complex regional pain syndrome: a systematic review and meta-analysis. Neurology.

[36] Brum ES (2022). Animal models of fibromyalgia: what is the best choice?. Pharmacol Ther.

[37] Liu Y (2021). Animal models of complex regional pain syndrome type I. J Pain Res.

[38] Finan PH (2013). The association of sleep and pain: an update and a path forward. J Pain.

[39] Vogel JW (2023). Connectome-based modelling of neurodegenerative diseases: towards precision medicine and mechanistic insight. Nat Rev Neurosci.

[40] Goebel A (2021). Passive transfer of fibromyalgia symptoms from patients to mice. J Clin Invest.

[41] Barbara G (2004). Activated mast cells in proximity to colonic nerves correlate with abdominal pain in irritable bowel syndrome. Gastroenterology.

[42] Sommer C (2018). Inflammation in the pathophysiology of neuropathic pain. Pain.

[43] Costigan M (2009). T-cell infiltration and signaling in the adult dorsal spinal cord is a major contributor to neuropathic pain-like hypersensitivity. J Neurosci.

[44] McDougall JJ (2006). Arthritis and pain. Neurogenic origin of joint pain. Arthritis Res Ther.

[45] Ramachandran R (2013). TRPM8 activation attenuates inflammatory responses in mouse models of colitis. Proc Natl Acad Sci U S A.

[46] Liu XJ (2014). Nociceptive neurons regulate innate and adaptive immunity and neuropathic pain through MyD88 adapter. Cell Res.

[47] Matsuda M (2019). Roles of inflammation, neurogenic inflammation, and neuroinflammation in pain. J Anesth.

[48] Pinho-Ribeiro FA (2018). Blocking neuronal signaling to immune cells treats streptococcal invasive infection. Cell.

[49] Scholzen TE (2004). Cutaneous allergic contact dermatitis responses are diminished in mice deficient in neurokinin 1 receptors and augmented by neurokinin 2 receptor blockage. FASEB J.

[50] Littlejohn G, Guymer E (2018). Neurogenic inflammation in fibromyalgia. Semin Immunopathol.

[51] Inyang KE (2019). The antidiabetic drug metformin prevents and reverses neuropathic pain and spinal cord microglial activation in male but not female mice. Pharmacol Res.

[52] Ray PR (2023). RNA profiling of human dorsal root ganglia reveals sex differences in mechanisms promoting neuropathic pain. Brain.

[53] Fan CY (2025). Divergent sex-specific pannexin-1 mechanisms in microglia and T cells underlie neuropathic pain. Neuron.

[54] Midavaine E (2025). Meningeal regulatory T cells inhibit nociception in female mice. Science.

[55] Zheng Q (2022). Synchronized cluster firing, a distinct form of sensory neuron activation, drives spontaneous pain. Neuron.

[56] Xie W Vascular motion in the dorsal root ganglion sensed by Piezo2 in sensory neurons triggers episodic neuropathic pain. Neuron.

[57] Wei Z (2019). Emerging role of Schwann cells in neuropathic pain: receptors, glial mediators and myelination. Front Cell Neurosci.

[58] Singhmar P (2020). The fibroblast-derived protein PI16 controls neuropathic pain. Proc Natl Acad Sci U S A.

[59] Hanani M, Spray DC (2020). Emerging importance of satellite glia in nervous system function and dysfunction. Nat Rev Neurosci.

[60] Bai Z (2024). Synovial fibroblast gene expression is associated with sensory nerve growth and pain in rheumatoid arthritis. Sci Transl Med.

[61] McLean SA (2005). Momentary relationship between cortisol secretion and symptoms in patients with fibromyalgia. Arthritis Rheum.

[62] Crofford LJ (1994). Hypothalamic-pituitary-adrenal axis perturbations in patients with fibromyalgia. Arthritis Rheum.

[63] Singh VP (2014). Advanced glycation end products and diabetic complications. Korean J Physiol Pharmacol.

[64] Edwards RR (2016). The role of psychosocial processes in the development and maintenance of chronic pain. J Pain.

[65] Smith ML (2021). Anterior cingulate inputs to nucleus accumbens control the social transfer of pain and analgesia. Science.

[66] Altawil R (2016). Remaining pain in early rheumatoid arthritis patients treated with methotrexate. Arthritis Care Res (Hoboken).

[67] Kamei T (2024). Unique electrophysiological property of a novel Nav1.7, Nav1.8, and Nav1.9 sodium channel blocker, ANP-230. Biochem Biophys Res Commun.

[68] Balanaser M (2023). Combination pharmacotherapy for the treatment of neuropathic pain in adults: systematic review and meta-analysis. Pain.

[69] Kehlet H (2006). Persistent postsurgical pain: risk factors and prevention. Lancet.

[70] Buchheit T (2012). Epigenetics and the transition from acute to chronic pain. Pain Med.

[71] Zorina-Lichtenwalter K (2016). Genetic predictors of human chronic pain conditions. Neuroscience.

[72] Ji RR (2018). Neuroinflammation and central sensitization in chronic and widespread pain. Anesthesiology.

[73] Takeshita Y, Ransohoff RM (2012). Inflammatory cell trafficking across the blood-brain barrier: chemokine regulation and in vitro models. Immunol Rev.

[74] Kesmati M, Torabi M (2014). Interaction between analgesic effect of nano and conventional size of zinc oxide and opioidergic system activity in animal model of acute pain. Basic Clin Neurosci.

[75] Jahangiri L (2013). Evaluation of analgesic and anti-inflammatory effect of nanoparticles of magnesium oxide in mice with and without ketamine. Eur Rev Med Pharmacol Sci.

[76] Franz-Montan M (2007). Liposome-encapsulated ropivacaine for topical anesthesia of human oral mucosa. Anesth Analg.

[77] Bean BP (2007). The action potential in mammalian central neurons. Nat Rev Neurosci.

[78] Bennett DL (2019). The role of voltage-gated sodium channels in pain signaling. Physiol Rev.

[79] Jones J (2023). Selective inhibition of Na_V_1.8 with VX-548 for acute pain. N Engl J Med.

[80] Price N (2017). Safety and efficacy of a topical sodium channel inhibitor (TV-45070) in patients with postherpetic neuralgia (PHN): a randomized, controlled, proof-of-concept, crossover study, with a subgroup analysis of the Nav1.7 R1150W genotype. Clin J Pain.

[81] Fetell M (2023). Cutaneous nerve fiber and peripheral Nav1.7 assessment in a large cohort of patients with postherpetic neuralgia. Pain.

[82] Kraus RL (2021). Na_v_1.7 target modulation and efficacy can be measured in nonhuman primate assays. Sci Transl Med.

[83] Kim JS (2024). Role of Na_V_1.7 in postganglionic sympathetic nerve function in human and guinea-pig arteries. J Physiol.

[84] Regan CP (2024). Autonomic dysfunction linked to inhibition of the Na_v_1.7 sodium channel. Circulation.

[85] Okuda H (2023). Reduced pain sensitivity of episodic pain syndrome model mice carrying a Nav1.9 mutation by ANP-230, a novel sodium channel blocker. Heliyon.

[86] Harrison C (2025). Vertex’s opioid-free drug for acute pain wins FDA approval. Nat Biotechnol.

[87] Kingwell K (2025). Na_V_1.8 inhibitor poised to provide opioid-free pain relief. Nat Rev Drug Discov.

[88] Zamponi GW (2016). Targeting voltage-gated calcium channels in neurological and psychiatric diseases. Nat Rev Drug Discov.

[89] Nair AS (2018). Ziconotide: indications, adverse effects, and limitations in managing refractory chronic pain. Indian J Palliat Care.

[90] Bauer CS (2009). The increased trafficking of the calcium channel subunit alpha2delta-1 to presynaptic terminals in neuropathic pain is inhibited by the alpha2delta ligand pregabalin. J Neurosci.

[91] Patel R, Dickenson AH (2016). Mechanisms of the gabapentinoids and alpha 2 delta-1 calcium channel subunit in neuropathic pain. Pharmacol Res Perspect.

[92] Wiffen PJ (2017). Gabapentin for chronic neuropathic pain in adults. Cochrane Database Syst Rev.

[93] Alles SRA, Smith PA (2021). Peripheral voltage-gated cation channels in neuropathic pain and their potential as therapeutic targets. Front Pain Res (Lausanne).

[94] Tsantoulas C, McMahon SB (2014). Opening paths to novel analgesics: the role of potassium channels in chronic pain. Trends Neurosci.

[95] Devulder J (2010). Flupirtine in pain management: pharmacological properties and clinical use. CNS Drugs.

[96] Kornhuber J (1999). Flupirtine shows functional NMDA receptor antagonism by enhancing Mg2+ block via activation of voltage independent potassium channels. Rapid communication. J Neural Transm (Vienna).

[97] Li C (2008). Analgesic efficacy and tolerability of flupirtine vs. tramadol in patients with subacute low back pain: a double-blind multicentre trial. Curr Med Res Opin.

[98] Naser SM (2012). Efficacy and safety of flupirtine maleate and tramadol hydrochloride in postoperative pain management—a prospective randomised double blinded study. J Indian Med Assoc.

[99] Michel MC (2012). Unexpected frequent hepatotoxicity of a prescription drug, flupirtine, marketed for about 30 years. Br J Clin Pharmacol.

[100] French JA (2023). Efficacy and safety of XEN1101, a novel potassium channel opener, in adults with focal epilepsy: a phase 2b randomized clinical trial. JAMA Neurol.

[101] Patapoutian A (2009). Transient receptor potential channels: targeting pain at the source. Nat Rev Drug Discov.

[102] Szczot M (2018). PIEZO2 mediates injury-induced tactile pain in mice and humans. Sci Transl Med.

[103] Murthy SE (2018). The mechanosensitive ion channel Piezo2 mediates sensitivity to mechanical pain in mice. Sci Transl Med.

[104] Qi L (2024). A mouse DRG genetic toolkit reveals morphological and physiological diversity of somatosensory neuron subtypes. Cell.

[105] Qian X (2023). Current status of GABA receptor subtypes in analgesia. Biomed Pharmacother.

[106] Zhuo M (2017). Ionotropic glutamate receptors contribute to pain transmission and chronic pain. Neuropharmacology.

[107] Witschi R (2011). Presynaptic alpha2-GABAA receptors in primary afferent depolarization and spinal pain control. J Neurosci.

[108] Solway B (2011). Tonic inhibition of chronic pain by neuropeptide Y. Proc Natl Acad Sci U S A.

[109] Nelson TS (2023). Alleviation of neuropathic pain with neuropeptide Y requires spinal Npy1r interneurons that coexpress Grp. JCI Insight.

[110] Shepherd AJ (2018). Macrophage angiotensin II type 2 receptor triggers neuropathic pain. Proc Natl Acad Sci U S A.

[111] Rice ASC (2021). Efficacy and safety of EMA401 in peripheral neuropathic pain: results of 2 randomised, double-blind, phase 2 studies in patients with postherpetic neuralgia and painful diabetic neuropathy. Pain.

[112] Kalliomaki J (2013). A randomized, double-blind, placebo-controlled trial of a chemokine receptor 2 (CCR2) antagonist in posttraumatic neuralgia. Pain.

[113] Donvito G (2018). The endogenous cannabinoid system: a budding source of targets for treating inflammatory and neuropathic pain. Neuropsychopharmacology.

[114] Castro J (2022). Olorinab (APD371), a peripherally acting, highly selective, full agonist of the cannabinoid receptor 2, reduces colitis-induced acute and chronic visceral hypersensitivity in rodents. Pain.

[115] Chang L (2023). Efficacy and safety of olorinab, a full agonist of the cannabinoid receptor 2, for the treatment of abdominal pain in patients with irritable bowel syndrome: results from a phase 2b randomized placebo-controlled trial (CAPTIVATE). Neurogastroenterol Motil.

[116] Whiting PF (2015). Cannabinoids for medical use: a systematic review and meta-analysis. JAMA.

[117] Mlost J (2020). Cannabidiol for pain treatment: focus on pharmacology and mechanism of action. Int J Mol Sci.

[118] Pertwee RG (2008). The diverse CB1 and CB2 receptor pharmacology of three plant cannabinoids: delta9-tetrahydrocannabinol, cannabidiol and delta9-tetrahydrocannabivarin. Br J Pharmacol.

[119] Zhang HB, Bean BP (2021). Cannabidiol inhibition of murine primary nociceptors: tight binding to slow inactivated states of Na_v_1.8 channels. J Neurosci.

[120] Zhang HB (2022). Cannabidiol activates neuronal Kv7 channels. Elife.

[121] Fisher E (2021). Cannabinoids, cannabis, and cannabis-based medicine for pain management: a systematic review of randomised controlled trials. Pain.

[122] Skrabek RQ (2008). Nabilone for the treatment of pain in fibromyalgia. J Pain.

[123] Cittadini E (2006). Effectiveness of intranasal zolmitriptan in acute cluster headache: a randomized, placebo-controlled, double-blind crossover study. Arch Neurol.

[124] Kuca B (2018). Lasmiditan is an effective acute treatment for migraine: a phase 3 randomized study. Neurology.

[125] Lederman S (2023). Efficacy and safety of sublingual cyclobenzaprine for the treatment of fibromyalgia: results from a randomized, double-blind, placebo-controlled trial. Arthritis Care Res (Hoboken).

[126] Giovannitti JA (2015). Alpha-2 adrenergic receptor agonists: a review of current clinical applications. Anesth Prog.

[127] Fink EA (2022). Structure-based discovery of nonopioid analgesics acting through the α_2A_-adrenergic receptor. Science.

[129] Ohashi N, Kohno T (2020). Analgesic effect of acetaminophen: a review of known and novel mechanisms of action. Front Pharmacol.

[130] Marks DM (2009). Serotonin-norepinephrine reuptake inhibitors for pain control: premise and promise. Curr Neuropharmacol.

[131] Sanchez-Robles EM (2021). Monoclonal antibodies for chronic pain treatment: present and future. Int J Mol Sci.

[132] Sevcik MA (2005). Anti-NGF therapy profoundly reduces bone cancer pain and the accompanying increase in markers of peripheral and central sensitization. Pain.

[133] Dakin P (2021). Efficacy and safety of fasinumab in patients with chronic low back pain: a phase II/III randomised clinical trial. Ann Rheum Dis.

[134] Dakin P (2019). The efficacy, tolerability, and joint safety of fasinumab in osteoarthritis pain: a phase IIb/III double-blind, placebo-controlled, randomized clinical trial. Arthritis Rheumatol.

[135] Lee KM (2020). CCL17 in inflammation and pain. J Immunol.

[136] Shin H (2023). The GM-CSF/CCL17 pathway in obesity-associated osteoarthritic pain and disease in mice. Osteoarthritis Cartilage.

[137] Nijjar JS (2025). Efficacy, safety and tolerability of GSK3858279, an anti-CCL17 monoclonal antibody and analgesic, in healthy volunteers and patients with knee osteoarthritis pain: a phase I, randomised, double-blind, placebo-controlled, proof-of-mechanism and proof-of-concept study. Ann Rheum Dis.

[138] Boyle Y (2024). Randomized, placebo-controlled study on the effects of intravenous GSK3858279 (anti-CCL17) on a battery of evoked pain tests in healthy participants. Clin Transl Sci.

[139] Ren K, Torres R (2009). Role of interleukin-1beta during pain and inflammation. Brain Res Rev.

[140] Mailhot B (2020). Neuronal interleukin-1 receptors mediate pain in chronic inflammatory diseases. J Exp Med.

[141] Fleischmann RM (2019). A phase II trial of lutikizumab, an anti-interleukin-1α/β dual variable domain immunoglobulin, in knee osteoarthritis patients with synovitis. Arthritis Rheumatol.

[142] Cohen F (2022). The arrival of anti-CGRP monoclonal antibodies in migraine. Neurotherapeutics.

[143] Sakloth F (2020). HDAC6-selective inhibitors decrease nerve-injury and inflammation-associated mechanical hypersensitivity in mice. Psychopharmacology (Berl).

[144] Krukowski K (2017). HDAC6 inhibition effectively reverses chemotherapy-induced peripheral neuropathy. Pain.

[145] Michelson D (2023). A randomized, double-blind, placebo-controlled study of histone deacetylase type 6 inhibition for the treatment of painful diabetic peripheral neuropathy. Pain Rep.

[146] Madigan V (2023). Drug delivery systems for CRISPR-based genome editors. Nat Rev Drug Discov.

[147] Dhuri K (2020). Antisense oligonucleotides: an emerging area in drug discovery and development. J Clin Med.

[148] Moreno AM (2021). Long-lasting analgesia via targeted in situ repression of Na_V_1.7 in mice. Sci Transl Med.

[149] Zhao X (2013). A long noncoding RNA contributes to neuropathic pain by silencing Kcna2 in primary afferent neurons. Nat Neurosci.

[150] Mohan A (2018). Antisense oligonucleotides selectively suppress target RNA in nociceptive neurons of the pain system and can ameliorate mechanical pain. Pain.

[151] Hoang DM (2022). Stem cell-based therapy for human diseases. Signal Transduct Target Ther.

[152] Jusop AS (2023). Development of brain organoid technology derived from iPSC for the neurodegenerative disease modelling: a glance through. Front Mol Neurosci.

[153] Chaudhuri O (2016). Hydrogels with tunable stress relaxation regulate stem cell fate and activity. Nat Mater.

[154] Michoud F (2021). Epineural optogenetic activation of nociceptors initiates and amplifies inflammation. Nat Biotechnol.

[155] Choi S (2020). Parallel ascending spinal pathways for affective touch and pain. Nature.

[156] Ibsen S (2015). Sonogenetics is a non-invasive approach to activating neurons in Caenorhabditis elegans. Nat Commun.

[157] Duque M (2022). Sonogenetic control of mammalian cells using exogenous Transient Receptor Potential A1 channels. Nat Commun.

[158] Perez-Sanchez J (2023). A humanized chemogenetic system inhibits murine pain-related behavior and hyperactivity in human sensory neurons. Sci Transl Med.

[159] Mathis A (2018). DeepLabCut: markerless pose estimation of user-defined body parts with deep learning. Nat Neurosci.

[160] Zhang Z (2022). Automated preclinical detection of mechanical pain hypersensitivity and analgesia. Pain.

[161] Bohnslav JP (2021). DeepEthogram, a machine learning pipeline for supervised behavior classification from raw pixels. Elife.

[162] Wiltschko AB (2020). Revealing the structure of pharmacobehavioral space through motion sequencing. Nat Neurosci.

[163] Weinreb C (2024). Keypoint-MoSeq: parsing behavior by linking point tracking to pose dynamics. Nat Methods.

[164] Lee J (2019). Machine learning-based prediction of clinical pain using multimodal neuroimaging and autonomic metrics. Pain.

[165] Shirvalkar P (2023). First-in-human prediction of chronic pain state using intracranial neural biomarkers. Nat Neurosci.

[166] Sharma N (2020). The emergence of transcriptional identity in somatosensory neurons. Nature.

[167] Renthal W (2020). Transcriptional reprogramming of distinct peripheral sensory neuron subtypes after axonal injury. Neuron.

[168] Tavares-Ferreira D (2022). Spatial transcriptomics of dorsal root ganglia identifies molecular signatures of human nociceptors. Sci Transl Med.

[169] Bhuiyan SA (2024). Harmonized cross-species cell atlases of trigeminal and dorsal root ganglia. Sci Adv.

[170] Pogatzki-Zahn EM (2021). A proteome signature for acute incisional pain in dorsal root ganglia of mice. Pain.

[171] Hanna R (2024). Proteomic analysis of dorsal root ganglia in a mouse model of paclitaxel-induced neuropathic pain. PLoS One.

[172] Jamieson DG (2014). The pain interactome: connecting pain-specific protein interactions. Pain.

[173] Jain A (2024). Nociceptor-immune interactomes reveal insult-specific immune signatures of pain. Nat Immunol.

[174] Wangzhou A (2021). A ligand-receptor interactome platform for discovery of pain mechanisms and therapeutic targets. Sci Signal.

[175] Parisien M (2024). Genome-wide association studies with experimental validation identify a protective role for B lymphocytes against chronic post-surgical pain. Br J Anaesth.

[176] Suri P (2018). Genome-wide meta-analysis of 158,000 individuals of European ancestry identifies three loci associated with chronic back pain. PLoS Genet.

[177] Catacutan DB (2024). Machine learning in preclinical drug discovery. Nat Chem Biol.

[178] Vamathevan J (2019). Applications of machine learning in drug discovery and development. Nat Rev Drug Discov.

[179] Bunne C (2024). How to build the virtual cell with artificial intelligence: priorities and opportunities. Cell.

[180] Mullard A (2016). Parsing clinical success rates. Nat Rev Drug Discov.

[181] Harrison RK (2016). Phase II and phase III failures: 2013-2015. Nat Rev Drug Discov.

[182] North RY (2019). Electrophysiological and transcriptomic correlates of neuropathic pain in human dorsal root ganglion neurons. Brain.

[183] Bartley EJ, Fillingim RB (2013). Sex differences in pain: a brief review of clinical and experimental findings. Br J Anaesth.

[184] Ahanonu B (2024). Long-term optical imaging of the spinal cord in awake behaving mice. Nat Methods.

[185] Wainger BJ (2015). Modeling pain in vitro using nociceptor neurons reprogrammed from fibroblasts. Nat Neurosci.

[186] Kim JI Human assembloid model of the ascending neural sensory pathway. Nature.

[187] Seah BC, Teo BM (2018). Recent advances in ultrasound-based transdermal drug delivery. Int J Nanomedicine.

[188] Berta T (2017). Targeting dorsal root ganglia and primary sensory neurons for the treatment of chronic pain. Expert Opin Ther Targets.

[189] Pons-Faudoa FP (2019). Advanced implantable drug delivery technologies: transforming the clinical landscape of therapeutics for chronic diseases. Biomed Microdevices.

[190] Heo K (2025). Non-muscle myosin II inhibition at the site of axon injury increases axon regeneration. Nat Commun.

[191] Li J, Mooney DJ (2016). Designing hydrogels for controlled drug delivery. Nat Rev Mater.

[192] Mitchell MJ (2021). Engineering precision nanoparticles for drug delivery. Nat Rev Drug Discov.

[193] Waghule T (2019). Microneedles: a smart approach and increasing potential for transdermal drug delivery system. Biomed Pharmacother.

[194] Hu M (2020). Micro/nanorobot: a promising targeted drug delivery system. Pharmaceutics.

[195] Binshtok AM (2007). Inhibition of nociceptors by TRPV1-mediated entry of impermeant sodium channel blockers. Nature.

[196] Tochitsky I (2021). Inhibition of inflammatory pain and cough by a novel charged sodium channel blocker. Br J Pharmacol.

[197] Dumanian GA (2019). Targeted muscle reinnervation treats neuroma and phantom pain in major limb amputees: a randomized clinical trial. Ann Surg.

[198] Knotkova H (2021). Neuromodulation for chronic pain. Lancet.

[199] Sun Y (2021). Mesenchymal stem cells-derived exosomes for drug delivery. Stem Cell Res Ther.

[200] Goudman L (2022). Virtual reality applications in chronic pain management: systematic review and meta-analysis. JMIR Serious Games.

[201] Paul AK (2021). Opioid analgesia and opioid-induced adverse effects: a review. Pharmaceuticals (Basel).

[202] Herzog RI (2003). Distinct repriming and closed-state inactivation kinetics of Nav1.6 and Nav1.7 sodium channels in mouse spinal sensory neurons. J Physiol.

[203] Faber CG (2023). Efficacy and safety of vixotrigine in idiopathic or diabetes-associated painful small fibre neuropathy (CONVEY): a phase 2 placebo-controlled enriched-enrolment randomised withdrawal study. EClinicalMedicine.

[204] Zakrzewska JM (2017). Safety and efficacy of a Nav1.7 selective sodium channel blocker in patients with trigeminal neuralgia: a double-blind, placebo-controlled, randomised withdrawal phase 2a trial. Lancet Neurol.

[205] Akopian AN (1996). A tetrodotoxin-resistant voltage-gated sodium channel expressed by sensory neurons. Nature.

[206] Renganathan M (2001). Contribution of Na(v)1.8 sodium channels to action potential electrogenesis in DRG neurons. J Neurophysiol.

[207] Cummins TR (1999). A novel persistent tetrodotoxin-resistant sodium current in SNS-null and wild-type small primary sensory neurons. J Neurosci.

[208] Huang J (2019). A novel gain-of-function Nav1.9 mutation in a child with episodic pain. Front Neurosci.

[209] Narahashi T (2008). Tetrodotoxin: a brief history. Proc Jpn Acad Ser B Phys Biol Sci.

[210] Hagen NA (2017). Tetrodotoxin for moderate to severe cancer-related pain: a multicentre, randomized, double-blind, placebo-controlled, parallel-design trial. Pain Res Manag.

[212] Lee M (2014). Z944: a first in class T-type calcium channel modulator for the treatment of pain. J Peripher Nerv Syst.

[213] Deeks ED (2019). Mirogabalin: first global approval. Drugs.

[214] Guo X (2024). GABA analogue HSK16149 in Chinese patients with diabetic peripheral neuropathic pain: a phase 3 randomized clinical trial. JAMA Netw Open.

[215] Qian K (2024). Discovery of a novel K_V_7.2/7.3 channels agonist for the treatment of neuropathic pain. Eur J Med Chem.

[216] Fleckenstein J (2013). Activation of axonal Kv7 channels in human peripheral nerve by flupirtine but not placebo — therapeutic potential for peripheral neuropathies: results of a randomised controlled trial. J Transl Med.

[217] Bienfait F (2023). Evaluation of 8% capsaicin patches in chemotherapy-induced peripheral neuropathy: a retrospective study in a comprehensive cancer center. Cancers (Basel).

[218] Landrum O (2023). Painful diabetic peripheral neuropathy of the feet: integrating prescription-strength capsaicin into office procedures. Pain Manag.

[219] Stevens RM (2019). Randomized, double-blind, placebo-controlled trial of intraarticular trans-capsaicin for pain associated with osteoarthritis of the knee. Arthritis Rheumatol.

[220] Szallasi A (2023). Resiniferatoxin: nature’s precision medicine to silence TRPV1-positive afferents. Int J Mol Sci.

[221] Aiyer R (2018). A systematic review of NMDA receptor antagonists for treatment of neuropathic pain in clinical practice. Clin J Pain.

[222] Houck DR (2019). NYX-2925, a novel N-methyl-D-aspartate receptor modulator: a first-in-human, randomized, double-blind study of safety and pharmacokinetics in adults. Clin Transl Sci.

[223] Smith MT (2013). Small molecule angiotensin II type 2 receptor (AT2R) antagonists as novel analgesics for neuropathic pain: comparative pharmacokinetics, radioligand binding, and efficacy in rats. Pain Med.

[224] Shepherd AJ (2018). Angiotensin II triggers peripheral macrophage-to-sensory neuron redox crosstalk to elicit pain. J Neurosci.

[225] Dutra RC (2013). The role of kinin B1 and B2 receptors in the persistent pain induced by experimental autoimmune encephalomyelitis (EAE) in mice: evidence for the involvement of astrocytes. Neurobiol Dis.

[226] Zhang ZJ (2017). Chemokines in neuron-glial cell interaction and pathogenesis of neuropathic pain. Cell Mol Life Sci.

[227] Pergolizzi JV (2019). The role of lofexidine in management of opioid withdrawal. Pain Ther.

[228] Obata H (2017). Analgesic mechanisms of antidepressants for neuropathic pain. Int J Mol Sci.

[231] Arnold LM (2012). Safety and efficacy of esreboxetine in patients with fibromyalgia: a fourteen-week, randomized, double-blind, placebo-controlled, multicenter clinical trial. Arthritis Rheum.

[232] Loudon P (2018). Demonstration of an anti-hyperalgesic effect of a novel pan-Trk inhibitor PF-06273340 in a battery of human evoked pain models. Br J Clin Pharmacol.

[233] Hirose M (2016). NGF/TrkA signaling as a therapeutic target for pain. Pain Pract.

[234] Neogi T (2022). Observed efficacy and clinically important improvements in participants with osteoarthritis treated with subcutaneous tanezumab: results from a 56-week randomized NSAID-controlled study. Arthritis Res Ther.

[235] Fallon M (2023). A randomized placebo-controlled trial of the anti-nerve growth factor antibody tanezumab in subjects with cancer pain due to bone metastasis. Oncologist.

[236] Markman JD (2020). Tanezumab for chronic low back pain: a randomized, double-blind, placebo- and active-controlled, phase 3 study of efficacy and safety. Pain.

[237] Schmelz M (2019). Nerve growth factor antibody for the treatment of osteoarthritis pain and chronic low-back pain: mechanism of action in the context of efficacy and safety. Pain.

[238] Bramson C (2015). Exploring the role of tanezumab as a novel treatment for the relief of neuropathic pain. Pain Med.

[239] Kelly KM (2019). Safety and efficacy of fulranumab in osteoarthritis of the hip and knee: results from four early terminated phase III randomized studies. Curr Med Res Opin.

[240] Yazici Y (2012). Efficacy of tocilizumab in patients with moderate to severe active rheumatoid arthritis and a previous inadequate response to disease-modifying antirheumatic drugs: the ROSE study. Ann Rheum Dis.

[241] Sebba A (2021). Pain: a review of interleukin-6 and its roles in the pain of rheumatoid arthritis. Open Access Rheumatol.

[242] Lamb YN, Deeks ED (2018). Sarilumab: a review in moderate to severe rheumatoid arthritis. Drugs.

[243] van de Putte LB (2004). Efficacy and safety of adalimumab as monotherapy in patients with rheumatoid arthritis for whom previous disease modifying antirheumatic drug treatment has failed. Ann Rheum Dis.

[244] Leung L, Cahill CM (2010). TNF-alpha and neuropathic pain—a review. J Neuroinflammation.

[245] Kremer J (2010). Golimumab, a new human anti-tumor necrosis factor alpha antibody, administered intravenously in patients with active rheumatoid arthritis: forty-eight-week efficacy and safety results of a phase III randomized, double-blind, placebo-controlled study. Arthritis Rheum.

[246] Smolen J (2009). Efficacy and safety of certolizumab pegol plus methotrexate in active rheumatoid arthritis: the RAPID 2 study. A randomised controlled trial. Ann Rheum Dis.

[247] Lipsky PE (2000). Infliximab and methotrexate in the treatment of rheumatoid arthritis. Anti-Tumor Necrosis Factor Trial in Rheumatoid Arthritis with Concomitant Therapy Study Group. N Engl J Med.

[248] Gjefsen E (2020). The effect of infliximab in patients with chronic low back pain and Modic changes (the BackToBasic study): study protocol of a randomized, double blind, placebo-controlled, multicenter trial. BMC Musculoskelet Disord.

[249] Mrozkova P (2016). The role of protease-activated receptor type 2 in nociceptive signaling and pain. Physiol Res.

[250] Dong M (2019). Botulinum and tetanus neurotoxins. Annu Rev Biochem.

